# Cardiac Biomarkers in Sports Cardiology

**DOI:** 10.3390/jcdd9120453

**Published:** 2022-12-11

**Authors:** Alexandru-Dan Costache, Maria-Magdalena Leon-Constantin, Mihai Roca, Alexandra Maștaleru, Răzvan-Constantin Anghel, Ioana-Mădălina Zota, Andrei Drugescu, Irina-Iuliana Costache, Adriana Chetran, Ștefana-Maria Moisă, Bogdan Huzum, Ovidiu Mitu, Carmen Cumpăt, Cezar Honceriu, Florin Mitu

**Affiliations:** 1 Department of Internal Medicine I, Faculty of Medicine, University of Medicine and Pharmacy “Grigore T. Popa”, 700115 Iasi, Romania; 2 Department of Cardiovascular Rehabilitation, Clinical Rehabilitation Hospital, 700661 Iasi, Romania; 3 Department of Cardiology, ”St. Spiridon” Emergency County Hospital, 700111 Iasi, Romania; 4 Department of Mother and Child Medicine-Pediatrics, “Grigore T. Popa” University of Medicine and Pharmacy, 700115 Iasi, Romania; 5 Department of Pediatrics I, “St. Maria” Clinical Emergency Hospital, 700309 Iasi, Romania; 6 Department of Morphofunctional Sciences II, Faculty of Medicine, University of Medicine and Pharmacy “Grigore T. Popa”, 700115 Iasi, Romania; 7 Department of Orthopaedics and Traumatology, “Sf. Spiridon” Emergency Hospital, 700111 Iasi, Romania; 8 Department of Management, Clinical Rehabilitation Hospital, 700661 Iasi, Romania; 9 Faculty of Physical Education and Sports, “Alexandru Ioan Cuza” University, 700115 Iasi, Romania

**Keywords:** athlete’s heart, sudden cardiac death, cardiac biomarkers

## Abstract

Sustained physical activity induces morphological and functional changes in the cardiovascular system. While mostly physiological, they can also become a trigger for major adverse cardiovascular events, the most severe of which are sudden cardiac arrest and sudden cardiac death. Therefore, any novel method which can help more accurately estimate the cardiovascular risk should be considered for further studying and future implementation in the standard protocols. The study of biomarkers is gaining more and more ground as they have already established their utility in diagnosing ischemic cardiac disease or in evaluating cardiac dysfunction in patients with heart failure. Nowadays, they are being implemented in the screening of apparently healthy individuals for the assessment of the cardiovascular risk. The aim of this paper is to gather published data regarding the measurements of cardiac biomarkers in athletes, i.e., troponins, myoglobin, CK-MB, NT-proBNP, and D-Dimers, and their potential use in the field of sports cardiology.

## 1. Introduction

In present-day society, there is an alarming growth in sedentary behavior. This is mainly due to an increase in screen device usage (e.g., computers, mobile phones, and televisions), either leisurely or for school or work purposes in conjunction with unhealthy diets based on processed meals and rich in saturated fats. This reduction in physical activity has led, over time, to a general increase in average weight in both children and adults, and to higher rates of obesity and adipose tissue mass. Moreover, this is associated with long-term increased cardiovascular risk, reduced quality of life and life expectancy, and higher incidences of ischemic heart disease or strokes among others. Therefore, a healthier lifestyle, including balanced meal plans and regular physical activity, is mandatory for cardiovascular disease prevention [1,2].

Additionally, recent studies have also highlighted the tendency towards isolation both at work and in private lives, which is also linked to less physical activity and higher rates of diseases. Therefore, active social lives are also part of a complete picture of a healthy lifestyle [3].

The beneficial effects of regular and moderate-intensity physical activity are well known and acknowledged in the scientific community, i.e., increased muscular mass, reduction in adipose tissue, lowering of body weight and BMI, chronic stabilization of glycemic values, and lipid profile. Apart from fewer occurrences of thromboembolic events, sustained physical activity leads to a slowing of cardiovascular aging and is associated with increased life expectancy and a higher quality of life. This evidence can be observed in groups of subjects at risk, such as older people and patients with a chronic disease, where a slowing of cardiovascular aging has been noted in studies over several years. The benefits of sports are also noted in rehabilitation programs in patients with known ischemic heart disease or chronic heart failure, where, through tailored programs, a return to previous capabilities is possible [4,5].

While knowing the overall benefits of physical activity, it is important to know the chronic changes it induces in the body. The term “athlete’s heart” embodies such changes which occur in persons who engage in chronic sports activities, and are essential in adapting to the increased and repeated hemodynamic stress. Morphologically, these include myocardial hypertrophy for increased contraction, and chamber dilation to accommodate a higher blood volume. Functionally, a higher ejection fraction to sustain the higher oxygen demand during effort and increased vagal tonus, which explains the lower resting heart rate frequencies of trained persons, have been observed [6].

However, while regular sports practice is regarded as being beneficial, it is not without its downside. All the above-mentioned cardiovascular adaptations can also become a trigger for certain events such as arrhythmias or worse. The awareness towards major adverse cardiovascular events (MACE) is growing lately, and the most feared are sudden cardiac arrest (SCA) and sudden cardiac death (SCD), which are being more often diagnosed. Since many such cases in young and apparently healthy young athletes have been widely reported in the media, both the American Heart Association (AHA) and the European Society of Cardiology have formulated recommendations for the screening of athletes and of persons engaging in physical activities. The 2020 European Society of Cardiology (ESC) guidelines on sports cardiology and exercise in patients with cardiovascular disease recommend as standard screening in athletes the use of patient’s history, physical examination, and a 12-lead standard ECG. Maximal exercise testing is recommended only in persons at high or very high risk, while biomarkers use is not mentioned. However, there is a lack of evidence that is being acknowledged. Given the growing concern and the need for more specific assessment methods, studies are focusing nowadays on additional techniques which could be implemented, including the use of biomarkers [7,8,9,10,11,12,13,14,15,16].

While biomarkers are being used on a larger scale as standard procedures in medicine and in cardiology, for example, troponin in the diagnosis of ischemic heart disease, in other cases, they are still being studied. In sports cardiology, the number of existing studies is few and on smaller samples, but they do highlight a potential use of biomarkers to assess the cardiovascular risk even in apparently healthy and trained people who engage in regular physical activity, be it leisurely or strenuous. Based on these studies and the research we have conducted so far, the aim of this review is to gather the most recent data and present the potential utility of cardiac biomarker measurements for cardiovascular risk assessment in athletes [17].

## 2. Vascular Adaptation in Athletes

The vascular system is also subjected to an adaptation process during chronic physical activity. These effects include improved nitric oxide (NO)-dependent endothelial function and arterial stiffening reduction. In younger athletes or older trained persons who still lead an active lifestyle, endothelial function is much improved, leading to a reduction in processes such as atherosclerosis development, being comparable to statin intervention. In addition, a reduced heart rate variability has been associated with stiffer vessels, secondary to endothelial and baroreflex dysfunctions. In older individuals, reduced vasodilator function is more frequent, which can be placed on the increasing degree of endothelial dysfunction and a constellation of risk factors such as metabolic syndrome, a sedentary lifestyle, and high levels of oxidative stress. All these, especially when combined, limit the vasodilating properties of NO and lead to increased vascular stiffness with increasing age [18,19].

Majerczak et al., however, showed in a study published in 2019 that included older former professional athletes who still lead a healthy lifestyle, even though not at the same training intensity, that the vascular aging process could not be slowed as compared to individuals of the same age who did not engage in sports or training regimens. Even with constant exercise, the previously mentioned benefits on the vascular system do not last throughout life, and age remains the primary determining factor in vascular aging [20].

## 3. Early Imaging Biomarker, Stressed Heart Morphology (SHM) as the Conjunctive Point of Determination in Combined Physiologic Exercise and Pathologic Stress Stimuli

Chronic hemodynamic stress is characterized by a remodeling process, that is segmentary, and begins in the basal septal region, progressing in time over the mid-apical segment. Such changes are usually considered physiological in elite athletes, but they may also hide a pathological substrate. Therefore, their interpretation is the key in distinguishing them from underdiagnosed pathological situations, such as the early stages of heart failure [21].

Investigating the impact of sports on the cardiac muscle, several studies have been conducted on athlete assessment using modern imaging techniques, such as cardiac magnetic resonance (CMR). For example, right ventricle (RV) evaluation proved to be crucial in assessing performance capabilities in trained athletes. RV stroke volume should increase during exercise due to decreased RV end-systolic volumes. In patients with cardiomyopathies or pulmonary valve disease, RV systolic function is reduced, which ultimately accounts for premature fatigue and a reduction in exercise capacity [22].

When studying the impact of physical exercise on myocardial hypertrophy using CMR, Lee et al. showed that in a cohort of healthy individuals, ventricular wall thickness above 13 mm was normal, as was asymmetrical wall hypertrophy as a physiological response to the effort. This information is useful in differentiating such adaptations from hypertrophic cardiomyopathy [23].

Imaging could differentiate between a physiological and a pathological hypertrophy, based on specific criteria. The most feared differential diagnosis is the hypertrophic cardiomyopathy (HCM). It is associated with frequent arrhythmias episodes and is the most frequent cause of SCD in young professional athletes. In echocardiography, a LV wall thickness between 13 and 18 mm is considered physiological in young adults who engage in sport activities. Therefore, one distinguishing criteria is the LV cavity, which is enlarged in the physiological hypertrophy, but not in HCM, where it can actually be reduced in size. Furthermore, the hypertrophy pattern may also advocate for HCM, although, as stated previously, an asymmetry can also be found in healthy individuals [24,25,26,27,28].

CMR offers precise measurements of the heart walls and chambers, and it can quantify late gadolinium enhancement (LGE), which is a marker of interstitial fibrosis, a common feature in HCM [28,29,30,31,32].

However, sometimes a clear diagnosis may not be easy to achieve solely through cardiac ultrasound or CMR. Therefore, a more specific method could be the increased levels of biomarkers. Firstly, biomarkers of interstitial fibrosis are the cleavage products of collagen synthesis and degradation. Secondly, HCM will be associated most of the time with higher levels of inflammatory markers (e.g., cytokins and interleukins), but most importantly with cardiac troponin T (cTnT). In this context, cTnT levels are reflective of myocardial necrosis. Another possibility is measuring the circulating levels of microRNAs (miR), particularly miR29a. They act as markers for cardiac hypertrophy and interstitial fibrosis and are also elevated in HCM [28,33,34,35,36,37,38,39,40,41].

Apart from HCM, several phenocopy conditions are also accompanied by myocardial hypertrophy (see Table 1). For the differential diagnosis between these and HCM, genetic testing or endomyocardial biopsy with histopathological examination is useful [28].

## 4. Cardiac Troponins

Cardiac troponins (cTn) regulate cardiac muscle contraction through their phosphorylation sites [42,43]. The physiological left ventricular hypertrophy encountered in athletes is characterized by increased Ca^2+^-dependent force production and in Ca^2+^-sensitivity, which, together with a reduction in cTn phosphorylation, explain the increased contractile function [44]. Alternatively, higher intensity and sudden physical stress in untrained persons does cause a rise in cTn serum levels, but normally not above the pathological threshold [45].

It has been shown that when used in conjunction with a stress test, high-sensitivity troponin (hs-cTn) levels reach their peak serum levels between 3 and 4 h after the test [46].

Multiple factors influence the troponin level variations after physical exercise. Apart from the type of activity, its duration and intensity, age, gender, and body composition also influence their rise, which may be used for tailoring training regimens [47].

Since they are the most specific markers of myocardial injury, detectable troponin levels should always be further assessed in athletes, regardless of the value, as they may be indicative of exercise-related myocardial micro injuries [48].

In 2018, during the North Sea Race, which is a 91 km leisure sport mountain bike race, participants had blood samples taken before, 3 h after, and 24 h after the event. The paper published in 2021 discussed the cTnI levels which were measured and, in all cases, peak values were at the 3 h mark, yet the inter-individual variability should be mentioned [49].

The particular patterns of troponin fluctuation, characterized by an early peak and rapid normalization, are not suggestive of myocardial necrosis but rather a secondary mechanism such as microvascular ischemia, a sudden surge of systemic inflammation, cardiac metabolic deficiency, or even renal function impairment. This is supported by Marshall et al., who concluded that troponin variations are more prevalent in persons subjected to short but high-intensity bouts of exercise rather than long-term light physical activities [50,51].

Since the beginning of the COVID-19 pandemic, one of the most frequently encountered complications was post-viral myocarditis. Young athletes who suffered from the disease were required to undergo a complete cardiac evaluation, which would assess the potential cardiac involvement, and guide their return to previous physical parameters. Firstly, a triad of ECG, trans-thoracic echocardiography (TTE), and cTn measurements were implemented, and if one of them would be abnormal, the suspicion for myocarditis was raised, and a cardiac magnetic resonance (CMR) was recommended [52,53,54,55,56].

Cardiac troponins also have strong negative predictive values, since low or undetectable hs-cTn levels would exclude an ischemic heart disease [57,58,59].

## 5. Myoglobin

Myoglobin is not only secreted by the myocardial cells, but also by the skeletal muscle cells. Therefore, it does rise in the case of myocardial injuries (acute coronary syndrome and myocarditis), but also in the case of skeletal muscle fatigue or disease (e.g., myositis). Although it is the earliest to be affected in the serum in case of an acute coronary syndrome (ACS), it is not specific unless other biomarkers’ levels also rise or electrocardiographic (ECG) changes appear, similarly to interpreting increases in CK-MB [60,61,62,63].

Apart from physical effort or pathological situations, myoglobin levels are also influenced by other factors, such as diet. For example, caffeine consumption through the cyclic AMP pathway in L6 myotubes causes an increase in measured serum myoglobin levels [64].

Despite this, its high sensitivity is useful in evaluating the response of skeletal muscles to physical activity (for example, athletes during high-intensity interval training), or the results of steroid intake on physical performance. This can be especially useful in creating individually tailored training programs for different physical activities [65,66,67].

A meta-analysis published in 2019 by Lam et al. and a randomized trial by Nieman et al. published in 2020 have both highlighted the beneficial effects of whey protein supplements on muscle recovery post intense exercise. The meta-analysis highlighted a reduction in circulating biomarker levels, including myoglobin, especially in the fourth and fifth recovery days. This is supported by the randomized trial, as positive ergogenic effects were noticed. These include an increase in essential amino acid levels, as well as a decrease in myoglobin and creatine-kinase values, compared to the control groups [66,67].

In 2022, Tota et al. published a study on 12 mixed martial arts fighters and their training regimens. Among other measurements, they assessed myoglobin levels. They noted increases in myoglobin levels, but described them as not being of cardiovascular origin, but rather secondary to the increased metabolic stress to which they were subjected, as in professionals, training and competitive periods are exhaustive. Additionally, they again underlined the potential use of myoglobin in creating tailored individual regimens for performance athletes. Naturally, the adaptations of the body during prolonged periods of physical stress (e.g., dehydration, weight loss, and muscle mass increase) should be taken into consideration [68].

Myoglobinuria can also be used in evaluating athletes as a marker of haptoglobin (Hpg) binding capacity. Since Hpg is depleted in athletes, free radical formation is increased, which, in turn, raises the susceptibility to inflammatory processes [69].

## 6. CK-MB

The MB isoenzyme of creatine-kinase (CK-MB) is more specific to the cardiac muscle than myoglobin. However, unless associated with a concomitant rise in cTn levels, it cannot predict the cardiovascular risk or be used to diagnose an ACS on its own. Due to its early dynamic, it is more useful in evaluating patients with recent-onset ACS or in those with an estimated glomerular filtration rate (eGFR) below 15 mL/min/m^2^ [70,71].

Total CK and its MB isoenzyme were among the first biomarkers to be studied in athletes. In earlier studies, they displayed a lower dynamic in sustained moderate-intensity activities, and a higher rise in muscle-related injuries, as highlighted by some studies as far back as the 1980s [72,73,74,75].

Symanski et al. published a study on professional swimmers where neither total CK, nor CK-MB had any significant rise. They took the blood samples before, 5 min post-, 6 h post-, and 24 h post-exercise and concluded that in trained persons, CK-MB should not rise unless a muscular trauma is produced [72].

This is similar to what Ketunen et al. explained as being a rise in total CK and CK-MB secondary to muscular trauma [73].

Jaffe et al. measured total CK and CK-MB levels in professional footballers after a match, and found a significant rise in their levels, given the intensity and duration of an entire football match. However, they also concluded that these were related to skeletal muscle release in the blood rather than myocardial injury [74].

Apple et al. went a step further and assessed the differences between the two main types of muscular fibers. In the human body, there are the fast-twitch fibers, with a fast explosive contraction and anaerobic metabolism, being more suited for short bouts of physical stress, and the slow-twitch fibers, with a lower intensity but sustained contraction and aerobic metabolism, more suited for sustained endurance efforts. They took samples from both types of fibers and found that total CK and CK-MB values were higher in the slow-twitch fibers and in endurance athletes. This can be explained by the sustained and prolonged release from muscles during such efforts [75].

CK-MB may also display a gender-related variability, as suggested by a study conducted by Chamera et al. on male and female football players. The protocol included running training sessions and blood samples taken before, immediately after, and 15 min after. Both groups showed an increase immediately after, though more notable in the female subjects, with a return to baseline at the 15 min time point [76].

## 7. NT-proBNP

The N-terminal prohormone of brain natriuretic peptide (NT-proBNP) is associated with aging and with hypertensive stress on the atria and atrial walls. Therefore, it has more use in elderly patients with known cardiac dysfunction [77,78,79]. Their levels may increase in non-trained people who engage in strenuous physical activities, but they are not significant for an ischemic cardiac disease, unless correlated with ECG changes or with a rise in cTn levels [45,80,81,82].

In a study conducted by Perrone et al. on athletes running a 50 km ultramarathon, both hs-cTnI and NT-proBNP levels increased their values in all subjects, and in 30 percent of them even beyond the pathological values. Even though the intensity and duration of the race should be considered, their interpretation is still subjected to future studies [83]. Similar results were also obtained by Banfi et al., who underline the role of NT-proBNP in the screening of both professional and amateur runners, and describe how such sudden increases in both NT-proBNP and cTnI are frequently encountered in athletes and should be treated as normal in the context of the cardiac adaptation to increased stress [84]. This is also comparable to a previous study by Banfi et al., who showed that in football and rugby players, increases in NT-proBNP post-effort are physiological and should be interpreted as such [85].

Another study assessed NT-proBNP in urine samples from rugby players before, immediately post-, and 36 h post-play in two consecutive games. The results also highlighted the increases in urinary levels of NT-proBNP. However, no correlation could be established with the external workload (e.g., number of impacts) [86].

Regarding those mentioned above, the interpretation of NT-proBNP in apparently healthy individuals and athletes is difficult, especially when differentiating benign findings from truly pathological ones. It has been stated that in athletes, the rise in BNP and NT-proBNP levels is independent of exercise-induced rises in immune markers (C-reactive protein, IL-6, cortisol) as they have in cardiovascular patients with associated systemic inflammation. Therefore, such increases, even in apparently healthy persons, should be considered physiological, if not exceeding the threshold value [87].

Additionally, at-rest values are also important in the overall interpretation since they would actually show a degree of cardiac dysfunction. If NT-proBNP rises to a pathological level only during exercise, but at rest it is undetectable or normal, then it could be interpreted as a physiological rise. However, if high levels are also present at rest, especially after a longer period of recovery, then a degree of cardiac dysfunction should be suspected, and further investigations are warranted. For example, when performing cardiac ultrasound and measuring the right ventricle (RV) strain on elite athletes, King et al. found a significant reduction in the isovolumetric acceleration (IVA) of the RV. However, they correlated it with reduced myocardial strain and normal levels of NT-proBNP, and concluded that these changes are due more to physiological adaptation processes during prolonged endurance exercise rather than to myocardial damage [88].

In patients with known cardiac dysfunction (e.g., post-myocardial infarction), NT-proBNP serial measurements can be used to assess the effect of cardiac rehabilitation programs. Jin et al. evaluated patients 12 months after they underwent a three- or four-week rehabilitation program and noted the dynamics of NT-proBNP values. Following the acute event, they were well above the reference value. However, following the program, they reversed back to normal values, proving how the rehabilitation programs induce a reduction in cardiac dysfunction following acute events [89].

## 8. D-Dimers

D-Dimers are markers of intravascular fibrinolysis. They are characterized by a high sensitivity but low specificity, as they may register higher levels in patients with diverse causes of coronary stenosis, such as atherosclerosis, but also in a number of other causes [90]. Rather than being traditional cardiac biomarkers, they are useful in venous thromboembolism, left atrium thrombosis in patients with atrial fibrillation, cardioembolic stroke, acute aortic dissection, or even in persons with implantable cardiac devices (see Table 2) [91,92].

In athletes, D-Dimers are used more to evaluate the thromboembolic risk and the effect certain physical activities or diets have on the hemostatic system. For example, as far back as 1995, it has been shown that steroid usage in weightlifters results in higher D-Dimer values compared to athletes who did not use any form of supplement. This is correlated with increased thrombin and plasmin production, and with cases of thrombosis in apparently healthy individuals with no previous atherosclerotic evidence [93].

Interestingly, it has been shown that physical activity can have a stimulating effect on both clot formation and fibrinolysis. Investigating markers of thrombin and fibrin formation and markers of fibrinolysis (including D-Dimers) during the recovery time, it was observed that, in the initial phase, all registered increased values. However, it was in the late stages that, while fibrinolysis declined, clotting activity persisted, suggesting a light tendency towards thrombosis. This was also independent of whether the subject suffered from activated protein C resistance [94,95].

Further, even if no cardiac disease is involved, D-Dimers still have a use in evaluating athletes, especially in those apparently healthy with no previous known illnesses who suffer from sudden fatigue. In 2010, Korsten-Reck et al. published a case report on two long-distance runners with a sudden and significant reduction in physical performance. The only altered laboratory finding was the D-Dimers value. Eventually, the diagnosis of pulmonary embolism was established. This is interesting because neither of them had any history of illness or thrombophilic risk factors [96].

Regarding effective value interpretation, the HemSter study showed that in a group of female athletes, the cut-offs were different than in the non-athletic population. For example, while white blood cell count (WBC), prothrombin time (PT), and activated partial thromboplastin time (APTT) were the same as in the general population, C-reactive protein (CRP) had a lower cut-off (<2.9 mg/L) and fibrinogen and D-Dimers were higher (1.9–4.4 g/L and 852 µg/L respectively). The higher D-Dimer values should therefore be considered with regard to higher cut-offs and not necessarily be interpreted as pathological [97].

## 9. Other Biomarkers with Potential Use in Athlete Evaluation

Apart from those already discussed in this review so far, there are others that have been tested on athletes and have shown potential in their assessments (see Table 3). For assessing chronic stress and fatigue, cortisol and testosterone were measured. A sudden increase in stress causes higher cortisol concentrations, inhibiting testosterone secretion. Therefore, the cortisol/testosterone ratio is a feasible tool [98].

In case of overtraining, the human body, and especially the skeletal muscles, releases higher levels of end products, which can then be measured. Lactate is increased during training sessions, and when its threshold is reached, it is usually associated with fatigue. Therefore, it is useful in assessing the duration of physical effort and intensity one can achieve. This is also true for total CK, which rises slower in trained individuals than in untrained individuals who engage in strenuous exercise or suffer physical traumas, similar to LDH. Usually, in medicine, creatinine is used to evaluate the renal function. However, its interpretation in athletes may vary given their larger muscles mass, diet, and the effort to which they are subjected. Ammonia is a marker of predominantly anaerobic metabolism in athletes, registering higher values during sports that involve mostly short bursts (e.g., sprinters), than in those engaging in constant long-duration activities (e.g., marathon runners). Further assessment can be made with measurements of urea and uric acid levels, which are related to protein or supplement-rich diets and protein energy substrate utilization during the competitive season, and proteins released during tissue damage as in excessive effort or injuries [98].

Hypoxanthyne also proved to be a strong predictor of athletic performance, since it is a product of purine degradation. Its usefulness is supported by the fact that several studies have highlighted its higher sensitivity as compared to classic markers such as lactate [99,100,101].

**Table 3:** Biomarkers used in physical activity assessment (Palacios et al. [98]).

Category	Normal range	References
Chronic stress and fatigue		[98,100,102]
Cortisol	5–25 mcg/dL	
Testosterone	300–100 ng/dL (men) 15–70 ng/dL (women)	
Markers of overtraining		
Lactate	0.8–1.5 mmol/L	
CK	55–170 U/L (men) 30–145 U/L (women)	
Creatinine	0.7–1.3 mg/dL	
Ammonia	15–45 mcg/dL	
LDH	105–333 U/L	
Uric acid		
Urea	5–7 mmol/L	
Hypoxanthine	0.4–1.8 mcmol/L	
Markers of cardiovascular risk		
Homocysteine	5–15 mcmol/L	
cTnI	<1.5 ng/mL	
cTnT	<0.1 ng/mL	
CK-MB	10–20 U/L	
Myoglobin	<110 ng/mL	
NT-proBNP	<125 pg/ml	
D-Dimers	<0.5 mcg/mL	
Markers of oxidative stress		
Protein carbonyls	0.3–0.36 nmol/mg	
Superoxide dismutase	165–240 U/mL	
Glutathione peroxidase	2.65–4.8 U/mL	
Markers of inflammation		
C-reactive protein	<0.9 mg/dL	
Interleukin-6	<16 pg/mL	
Leukocytes	4500–11,000/mm^3^	

Hyperhomocysteinemia is considered a cardiovascular risk factor; therefore, measuring homocysteine levels in athletes can help evaluate their risk profile. In addition, it is generally accepted that constant, regular, and low–moderate intensity activities help reduce homocysteine levels, while in high-intensity sports, its levels tend to rise [98].

During physical activity, oxidative stress increases proportionally with the intensity and duration. Malondialdehyde (MDA), a marker of oxidative degradation of the cell membrane, and protein carbonyls (PC), markers of protein oxidative degradation, are increased, especially in untrained individuals. However, there is also an increase in antioxidant enzyme levels, to counterbalance the release of reactive oxygen species (ROS) [98].

Inflammation markers (i.e., C-reactive protein, interleukin-6, and leukocytes) show a tendency towards increasing in untrained persons or at the beginning of training periods, followed by a reduction with time or in trained persons. This is suggestive of the adaptive process of the immune system toward chronic exposure to constant physical stress [98].

## 10. The Current Place of Biomarkers in Sports Cardiology

Given their sensitivity and specificity, laboratory explorations and the study of biomarkers are being implemented both for diagnostic and screening purposes [103]. Mahanty et al. did conclude that cTn, BNP, and hypoxanthine have a role in assessing the cardiovascular function when subjected to physical activity. While acknowledging that larger studies are warranted, they recommend future implementation of biomarkers as they offer valuable information such as better understanding of the cardiovascular stress during physical activity, better surveillance of patients, or exercise-related events [104].

It is acknowledged that intense physical activity would determine an increase in serum biomarkers levels, but an exact tendency or behavior is yet to be discovered. Many results from different studies show contradictions, and this causes an issue with their interpretation. Sometimes biomarkers do not show any dynamic. In other cases, they rise but are still within normal limits, while in other cases, they may also exceed the pathological threshold even in apparently healthy individuals [105].

For example, while cTnT, hs-cTnT, BNP, NT-proBNP, and D-dimers levels are influenced by physical activity, this can actually interfere with their interpretation in an emergency setting when a diagnosis of an acute coronary syndrome, pulmonary embolism, or acute heart failure is suspected, as shown by a meta-analysis published in 2015 by Sedaghat-Hamedani et al. and supported by a previous study conducted by Smith et al. in 2004 [106,107].

With regards to the five discussed biomarkers and their variations upon physical stress, we have conducted a study on 19 football players who underwent a cardiopulmonary testing. We took the blood samples before the test to determine the resting values and 3 h after they reached the peak load for the post-effort measurements. The 3 h interval was chosen to allow the biomarkers time to reach detectable blood values. NT-proBNP and troponin levels were undetectable in all athletes both at rest and post-effort. This is explainable as neither of them suffered from any form of cardiac dysfunction (therefore explaining the lack of detectable NT-proBNP samples), nor did any of them develop ischemic changes during the exercise, which would otherwise show higher troponin values. However, a few correlations were established between the variations of the D-Dimers, CK-MB, and myoglobin levels and the parameters of the CPET [108].

This is comparable to a study conducted by Hosseini et al. on professional footballers during a full match. They concluded that after a 90 min football match, players’ serum cTnI and NT-proBNP levels increased and were detectable in samples taken 24 h later, yet never at pathological values. Compared to our study, this could be explained by the duration and intensity of a full-length competitive game, which is significantly higher than a CPET evaluation, yet the question of interpretation rises again, despite them being within a normal range [109].

Usually, CK-MB and myoglobin would be the most sensitive markers related to physical activity due to them also being influenced by skeletal muscle stress. When measured immediately after, at the 24 h, 48 h, and 72 h time points following a high-intensity intermittent running protocol, myoglobin would increase faster and to higher values, with both returning to their baselines at the 24 h mark. Further, when studying a 12-day training period group, samples were taken before, on the 6th day, and on the 12th day. Myoglobin peaked on the 12th day while CK-MB peaked on the 6th day [110,111]. They also showed significant results when studying the effects of steroid intake, especially whey protein supplements, where an increase in essential amino acid levels and decreases in myoglobin and CK values were noted [65,66].

As Park et al. concluded in 2019, intense physical activity causes an increase in cTnT, CK-MB, and myoglobin. While CK-MB and myoglobin levels can be attributed to skeletal muscle damage, the interpretation of cTnT increase is still to be discussed [112]. This can be discussed in conjunction with the study by Çakir et al., who found that post-exercise cold water immersions lead to lower cTnT values, but do not influence myoglobin levels [113].

Apart from the cardiac biomarkers, the immune biomarkers also show potential in sports medicine, especially with regard to the chronic adaptation of the body to sustained physical activity. Their general tendency is to have an initial rise when the period of training begins, followed by a plateau or even a return to baseline values, as the effort period continues for a few weeks regularly, as seen in whole blood count (WBC) or interleukins [114].

Cardiac and respiratory rehabilitation programs could also benefit from the combination of CPET and serum biomarker measurements in the dynamic evaluation of patients. This is supported by Wang et al., who conducted a study on chronic heart failure (CHF) patients. It has been noted that the cardio-respiratory rehabilitation programs induce a recovery in heart and lung functions in these patients, which was observed in ultrasound parameters (e.g., a reduction in left ventricular end-diastolic volumes and increases in left ventricular ejection fraction and stroke volume), and CPET parameters (e.g., increase in maximum oxygen consumption—peak VO_2_ and oxygen consumption at the anaerobic threshold—VO_2_@AT). Further, increased performance during the 6 min walking test was noticed, as were lower levels of blood NT-proBNP, troponin I, and C-reactive protein. These data were also correlated with questionnaires such as the Minnesota living with heart failure questionnaire (MLHFQ), the self-rating anxiety scale (SAS), and the self-rating depression scale (SDS) scores, which registered lower values than before the initiation of the rehabilitation programs [115].

Apart from the physical effort itself, these biomarkers are also useful in assessing the impact of certain recovery techniques. For example, Banfi et al. concluded that whole-body cryotherapy caused a mild increase in serum NT-proBNP levels, yet still within the normal limits, while cTnI levels were not influenced. This highlights the safety of this technique as a recovery tool following intense exercise [116].

## 11. Conclusions

The use of biomarker measurements is growing in medicine as they offer valuable information. This is also applicable in the evaluation of athletes. Intense strenuous exercise bouts induce increases in their levels even in healthy persons, but their interpretation with regard to the cardiovascular risk is still being discussed. If their levels do not reach the pathological threshold, they are considered benign changes and warrant no further investigation. However, if they exceed that threshold, they would require further investigations and pre-competitive screening. This may not be applied to troponin, where each detectable value should be considered a warning sign, even if below the threshold. Given how CK-MB, myoglobin, and D-Dimer levels are also influenced by the skeletal muscles or by other diseases, cardiac troponins and NT-proBNP are more useful for pure cardiovascular assessment. Standardized protocols and further studies on larger groups are needed in order for them to become fully implemented.

## Figures and Tables

**Table 1:** Phenocopy conditions mimicking HCM (Marian et al. [28]).

Phenocopy Condition	References
AMPK mediated glycogen storage	[28]
Amyloidosis	
Anderson–Fabry disease	
Danon disease	
Friedreich ataxia	
Kearns–Sayre syndrome	
Myotonic dystrophy	
Neimann–Pick disease	
Noonan/LEOPARD syndromes (Rasopathies)	
Pompe disease	
Refsum disease	

**Table 2:** Pathological conditions with increased D-Dimers levels (Almorad et al. and Miller et al. [91,92]).

Conditions	References
Coronary atherosclerosis	[91,92]
Venous thrombosis	
Pulmonary embolism	
Atrial fibrillation with atrial thrombus	
Cardioembolic stroke	
Acute aortic dissection	
Implantable cardiac devices	

## Data Availability

Not applicable.
